# Exploring Meal Provision and Mealtime Challenges for Aged Care Residents Consuming Texture-Modified Diets: A Mixed Methods Study

**DOI:** 10.3390/geriatrics7030067

**Published:** 2022-06-15

**Authors:** Xiaojing Wu, Lina Yousif, Anna Miles, Andrea Braakhuis

**Affiliations:** 1 Department of Nutrition, Faculty of Medical and Health Sciences, University of Auckland, Auckland 1023, New Zealand; lyou561@aucklanduni.ac.nz (L.Y.); a.braakhuis@auckland.ac.nz (A.B.); 2 Department of Speech Science, School of Psychology, University of Auckland, Auckland 1023, New Zealand; a.miles@auckland.ac.nz

**Keywords:** dysphagia, aged care, texture-modified diet, mealtime

## Abstract

Dysphagia has become more prevalent with age. Thus, the demand for texture-modified diets (TMDs) has increased. While the nutritional perspectives have been studied, the provision of TMDs and mealtime practice has received less attention. This study aimed to explore the TMD provision and mealtime challenges of residents requiring TMDs in aged care facilities. The study was conducted across five aged care facilities using a mixed methods design involving 14 TMD menu audits by a foodservice dietitian, 15 mealtime observations, and semi-structured interviews with residents and staff (*n* = 18). TMD menus failed to meet all nutrition requirements and foodservice and clinical standards based on the dietitian NZ foodservice and nutrition audit tool. A content analysis offered three main themes: (1) Foodservice production. Inconsistent quality and meal portions were observed. The variety, choice, and portion size of TMDs required improvement based on the residents’ preferences; (2) Serving procedures. There was a lack of standardisation of meal distribution and feeding assistance; and (3) Dining environment. The dining room set-up varied across facilities, and residents expressed different preferences towards the dining environment. There is a need to improve staff awareness of mealtime consistency and optimise feeding assistance. The dining environment should be individualised to accommodate residents’ psychosocial needs. Standardised policies and continuous training can facilitate quality mealtime implementation.

## 1. Introduction

The rapid ageing population is a common global trend in developed countries, and this has a considerable impact on healthcare providers, including increased demand for residential aged care services [1]. Ageing comes with a number of physiological changes, and dysphagia (difficulty swallowing) has become a prevalent condition in aged care facilities due to comorbidities, muscle weakness, or tooth decay [2,3]. Dysphagia and chewing difficulties can compromise mealtime safety, efficiency, and enjoyment and, in turn, appetite and dietary intake, consequently increasing the risk of malnutrition [4,5,6]. In order to ease chewing and swallowing, it is a common practice in aged care facilities to provide texture-modified diets (TMDs) to residents with dysphagia, poor dentition, or cognitive–behavioural eating impairments [7,8]. Recent multi-centre studies reported that 27–47% of aged care residents required TMDs [9,10,11]. To obtain a softer and smoother texture, TMDs are often prepared by cutting the food into small pieces, tenderising, mincing, mashing, and blending with liquid. Sometimes, thickened fluids are routinely prescribed for dysphagic patients in order to slow the process of swallowing and avoid the incidence of aspiration [12]. Thickened fluids are often prepared with commercial thickeners to increase the viscosity. The International Dysphagia Diet Standardisation Initiative (IDDSI) framework has been adopted by food industry, foodservice, speech-language therapists and dietitians worldwide. The IDDSI defines TMDs into five levels and thickened fluids into four levels: level 7—easy to chew/regular; level 6—soft and bite-sized; level 5—minced and moist; level 4—pureed/extremely thick; level 3—liquidized/moderately thick; level 2—mildly thick; and level 1—slightly thick. Although TMDs are frequently used, there are still challenges in nutritional quality, palatability, standardization, and patient acceptability, which can lead to food refusal and a reduction in oral intake [8,13]. A previous review confirmed the suboptimal nutritional status in TMD consumers [14].

The high prevalence of malnutrition is one of the most frequently stated concerns in older adults, especially in settings where residents have a higher level of dependence [6,15]. Up to 30% of the residents in long-term and rehabilitation care institutions were malnourished [15]. Impaired function, dementia, and dysphagia are all known to be factors associated with malnutrition in aged care residents [16,17]. Considering the nutritional vulnerability of older adults, maintaining an optimal nutritional status is a key aspect of healthy ageing [18]. Despite the challenge of increasing rates of malnutrition in aged care, we should focus on the modifiable factors, such as oral health, social support, meal access, eating dependency, physical activity, food flavour, and appeal [19,20].

The nutrition implications of TMDs have been addressed in several studies, yet few studies have explored the foodservice production and mealtime experience associated with TMDs in residential aged care [14,21,22]. In addition to the nutritional quality of TMDs, food choices, staff availability, the dining environment, foodservice operation, and resident satisfaction should all be considered for optimum nutrition [23,24,25,26]. It has been widely observed that residents’ enjoyment of mealtimes is associated with their quality of life and psychological well-being [27,28]. Therefore, our study focused on the mealtime experience of aged care residents with dysphagia. The purpose of this exploratory study was to capture and describe residents’ mealtime challenges with TMDs post-IDDSI implementation, including the quality of TMD menus, the food options provided by the foodservice, foodservice operation, mealtime assistance, and the dining environment.

## 2. Methods

**Figure 1:**
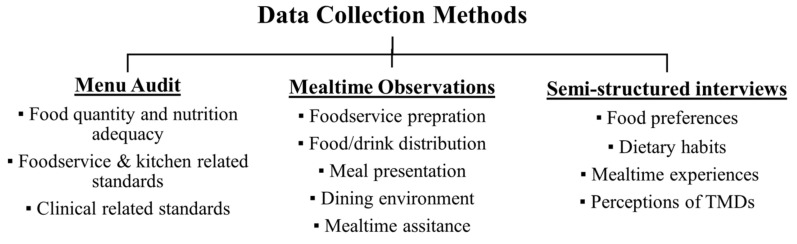
The data collection methods used in the study and the information collected from each method. TMDs = Texture-Modified Diets.

A cross-sectional mixed methods study was conducted, consisting of quantitative assessment tools and qualitative observations and interviews. In order to comprehend the complex circumstances, mealtime observations were used to gather information regarding behaviours, actions, and interactions [29]. Observations can provide deeper insights regarding the use of TMDs and residents’ experience by involving older people with communication or cognitive difficulties. While these unstructured mealtime observations allowed researchers to identify challenges in TMD provision, standardised menu audits counted the frequency of the issues observed, and the semi-structured interviews provided deeper perceptions and opinions about the issues. This triangulated approach allowed the researchers to verify the observations and gain a more comprehensive understanding of foodservice practices [30,31]. Figure 1 demonstrates the three data collection methods used in this study. Ethics approval was received from University of Auckland Human Participants Ethics Committee (023048) on 28 June 2019. Data collection was carried out from June 2020 until March 2021.

### 2.1. Participants

The study was conducted as part of a larger research project on post-IDDSI implementation evaluation in aged care facilities exploring nutrition intake, mealtime challenges, TMD compliance to IDDSI standards, and staff knowledge of the IDDSI framework before and after implementation. The parent project recruited five aged care facilities through convenience sampling, which included a range of small and large facilities with various foodservice systems [11]. In qualitative research, such sample selection is considered an appropriate and common approach to exploring opinions and identifying tentative hypotheses to be investigated in future studies [32]. All facilities had previously received IDDSI implementation. Facility managers gave their written consent to access the facility and approach staff to gather data. Consents were given for observing mealtime and foodservice practice and taking non-participants’ photographs. Informed consent was obtained from participants. The eligibility criteria required individuals to have received TMDs or be involved with TMD provision (including both foodservice and clinical departments).

### 2.2. Data Collection

Data were collected by two dietitian researchers (a registered dietitian with foodservice experience and a student dietitian). The data collection included daily observations of people, mealtime settings, activities, behaviour, interactions, staff roles, facility policies, the text of conversations, and photographs of meals/snacks/oral nutritional supplements (ONS) and dining room environments [32]. The qualitative data collected were systematically recorded as field notes and uploaded onto a confidential cloud drive at the end of each visit.

#### 2.2.1. Menu Audit

The dietitian researcher conducted menu audits using the Dietitians NZ Foodservice and Nutrition Audit tool 2020, which is an evidence-based tool created for aged care [33]. Only relevant menu audit and onsite audit sections related to the research questions have been included here, which consisted of the food quantity/nutrition adequacy of the menu, foodservice and kitchen-related procedures and policies, and clinic-related standards (Supplementary File S1). The facilities were requested to provide a copy of their current TMD menu with all seasonal cycles, a list of TMD recipes, and details of TMD resident dietary requirements. At mealtimes, foodservice managers prepared sample meals and provided documents related to kitchen standards and polices. Foodservice managers were also interviewed during the audit to clarify questions not addressed in the documentation, including routine practice, food options, and dietitian involvement in menu planning.

#### 2.2.2. Mealtime Observations

Mealtime observations were scheduled over three to four days of the week to ensure the data were comprehensive and representative [32]. The researchers conducted the observations at breakfast, morning tea, lunch, afternoon tea, and dinner on each of the days. The researchers also visited the foodservice prior to mealtime to weigh the portion sizes, check the menu accuracy, and observe meal preparations. We observed the foodservice staff, nurses, and healthcare assistants (HAs) during mealtime and recorded observations on the mealtime observation sheet (Supplementary File S2), including the preparation and distribution of texture-modified meals/drinks, snacks, and supplements, the dining room environment, and mealtime assistance. The dining room and meal service were photographed to ensure consistency in analysis. To minimise performance bias, the residents and staff were aware that nutrition intake data were being collected for the parent project through plate wastage observations, but they were unaware the researchers were also collecting data about the mealtime experience.

#### 2.2.3. Semi-Structured Interviews

The student dietitian conducted semi-structured interviews with residents consuming TMDs and key workers producing or serving TMDs (foodservice staff, nurses, and HAs). The interviews consisted of simple open-ended questions focusing on the residents’ preferences and consumptions, mealtime experience, and any thoughts on the TMDs. Interview prompt questions were reviewed by co-investigators (two experienced registered dietitians and a speech-language therapist with expertise in aged care and qualitative research). Staff and residents were selected through purposive sampling, which aimed to sample a group of people who have valuable experience in TMDs. Upon verbal consent, staff were interviewed in a private location to preserve confidentiality. Residents were interviewed in their room after mealtimes to provide context. Approximately 10 min were spent in the interview, which was digitally recorded. Hand-written notations were made on field notes to highlight key points. The purpose of the interview was to gain in-depth insight into the issues rather than seeking representation. Therefore, interviews continued until saturation was reached, when no more new themes or issues were raised by the participants [32].

### 2.3. Data Analysis

**Figure 2:**
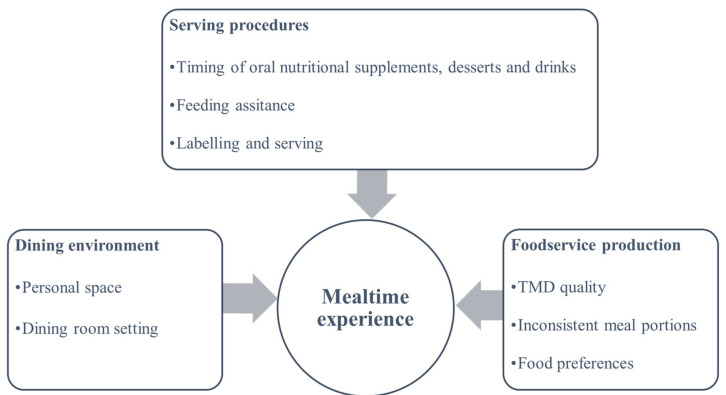
Final conceptual diagram demonstrating the three main themes and eight subthemes associated with mealtime challenges.

A descriptive analysis of all explanatory variables from audits was conducted and presented as a percentage of compliance and frequency counts. The digital recordings were transcribed by the interviewer and reviewed by a second researcher. The collected qualitative data (transcriptions and field notes) were then coded and analysed using conventional content analysis in NVivo 12 (QSR International, Melbourne, Australia) [34]. Analyses were guided by Kaid’s classic seven-step process, aiming to discover the underlying meanings of the content related to the research questions [35]. To capture the key concepts and identify the potential main themes, the researcher who completed the transcription immersed in the data by reading repeatedly and highlighting the keywords [36]. Subthemes were then developed to elaborate the main themes. Two other authors then reviewed the themes and discussed the potential themes with all authors to finalise the refined themes. The final conceptual diagram was developed to illustrate the relationships between the themes (Figure 2).

## 3. Results

There were 425 residents living in the five facilities. TMDs and thickened fluids were required by 20% (*n* = 85) and 5% (*n* = 22) of the residents, respectively. All participants requiring thickened fluids were on TMDs, mostly on pureed diets (64%). Overall, the prevalence of pureed diet consumers was the highest (9%), followed by soft and bite-sized (8%). Three facilities had a higher demand for pureed diets, while two other facilities had a higher demand for soft and bite-sized diets. Only three facilities had a requirement of minced and moist diets. The facilities ranged in size from 54- to 153-bed capacity, and all had an occupancy rate of 80%. Rest-home- and hospital-level care were offered in all five facilities, while dementia-level care was also offered in one facility. At the time of data collection, only one resident in the dementia unit was on TMDs. Detailed facility characteristics have been reported previously in the parent study [11]. There was one kitchen at each facility where meals were prepared. All facilities had access to dietitians and received IDDSI implementation six months prior to this study. Four facilities used cook-fresh systems to prepare level 6 soft and bite-sized diets and commercial pre-packaged meals for level 5 minced and moist and level 4 pureed diets, while Facility 4 used a cook-fresh system for all levels of TMDs. Commercial meals were shaped in silicone moulds beforehand and kept in the freezer. Each meal was plated with moulded protein and vegetables, then heated up in a steam oven prior to mealtime. Freshly made TMDs were served from bulk meal trolleys in each dining room.

A total of 18 staff and 8 residents were interviewed, including 5 foodservice staff, 4 nurses, and 9 HAs. Individual participants were recruited from the parental study. Half of the staff had 1–5 years of experience working in aged care facilities and with TMD provision.

Further detailed participant characteristics can be found in previous publications [37,38].

Based on the qualitative analysis, a conceptual diagram emerged, including three main themes contributing to mealtime challenges: (1) foodservice production, (2) serving procedures, and (3) the dining environment (Figure 2).

### 3.1. Foodservice Production

#### 3.1.1. TMD Quality

The menu audit results are shown in Table 1. A similar performance was detected in each audit objective, resulting in the similar overall performance of each facility. The highest compliance was found in foodservice and kitchen-related standards. Detailed non-compliant objectives are listed in Table 2. TMDs were provided at three levels in all facilities on a four-week cycle and seasonal menu. The foodservice managers reported the menu had been audited by dietitians annually. All facilities failed to meet all nutritional requirements recommended by the guidelines [39,40,41,42]. Food fortification has been implemented in all facilities except one (Facility 4). The commercial-packaged TMDs were pre-fortified with pea protein and freshly made meals were fortified with cream or full-cream milk powder.

Several concerns were raised regarding the palatability of texture-modified meals, including inconsistent texture, bland taste, limited variety, and poor appearance. As observed, residents on soft and bite-sized meals complained of tough meats and vegetables to the staff. An HA reported a similar issue that some residents on pureed diets found the creaminess of the meat and vegetables to be off-putting. A malnourished resident on soft and bite-sized meals refused to have his meals and sent it back to the kitchen, as he complained that he was unable to eat it due to swallowing difficulties. In response to the nurse’s suggestions on changing meal textures or taking ONS, he expressed that he disliked the taste of other meal textures or ONS. Other residents reported not liking the pureed meals and stated:

*“I miss home-cooked meals, the meals taste different here… And they don’t go well in my stomach”. *

*(Resident 03)*

*“All the meals are the same, there is no variety”. *

*Resident (04)*

We also noted the lack of alternative options for vegetarian/vegan TMDs. Although foodservice staff were aware of special diets and cultural food, there was a lack of standardisation in routine preparation. Vegetarian/vegan menus were not listed for pureed and minced and moist diets.

*“At both lunch and dinner, protein component was not offered to a resident who was on a vegetarian pureed diet, but only vegetable and carbohydrate portions”. *

*(Observation)*

In terms of presentation, four facilities used moulds for pureed and minced and moist meals. Some facilities served moulded meals for both lunch and dinner, while others only used the moulds when foodservice staff had time to prepare them. Although foodservice managers agreed that the application of moulding enhanced the aesthetics of the TMD meals, there were several challenges to implementing moulds in their kitchen, including limited space in the chiller and steamer, inconsistent TMD meal numbers, and a lack of time for preparation. One facility attempted to improve the appearance of texture-modified snacks by piping, enhancing the colour, and creating visually appealing plating. In fresh cooked TMDs, the major issue was the apparent lumps in pureed meals, indicating an inappropriate consistency.

*“The old meals (before introducing moulding) didn’t look very nice; I probably won’t eat it”. *

*(HA 04)*

*“They (moulded meals) look like the real sausages, peas and corns, but sometimes I still can’t tell what meat it is”. *

*(Resident 05)*

Foodservice staff and HAs both reported that the introduction of moulds has significantly improved residents’ food consumption. This was apparent based on several observations during audit visits. However, there were still some issues raised with the serving of TMD meals. Based on our observations, the consumptions of residents with cognitive impairment who required feeding were not significantly affected by moulded meals. Another concern raised by the staff was that the moulded shape did not always hold once served.

*“Even though the meal component looks like the actual food, the texture changes. It becomes flattened once the resident takes a spoonful off the plate. After they take a spoonful, the resident would realise that it is not the actual food and disappoint them”. *

*(HA 01)*

Changes in texture and appearance were likely caused by the temperature change. The meals were often delivered from the kitchen to the table in open air or were left uncovered once served.

*“Dinner meals served uncovered, scoops became flattened and looked unappetising”. *

*(Observation)*

#### 3.1.2. Inconsistent Meal Portions

There were notable differences when it came to the meal portions offered among the facilities and between the rest-home- and hospital-level care within a facility, especially with fruits and vegetables. Based on the audits, the portions of fresh fruit were limited. During the interview, residents confirmed that they did not always have access to fruits, but juice was often offered. Foodservice commonly prepared a meal with one portion of protein, one portion of carbohydrate, two portions of vegetables, and topped up with thickened gravy. There was a lack of routine provision of high-fibre foods at every meal and mid-meal, particularly whole grains. The staff served different portions to residents based on their knowledge of residents’ eating habits rather than confirming residents’ orders before mealtime. Residents were not asked whether they wanted extra portions during or after finishing their meals.

We noticed some residents had less than one portion of vegetables on their plate, but the staff reported they were served according to their preferences. When we interviewed the residents, some indicated that they preferred some vegetables over the others, but they were not told what would be served that day.

*“I am not a fan of broccoli; they are so plain. I like tomatoes, but they don’t have it very often and I don’t know what I’m getting” *

*(Resident 01)*

Furthermore, when meals were served from bulk containers or there were multiple staff serving during mealtimes, variations in serving portions were observed. We also noted a lack of awareness and communication between foodservice staff and HAs for appropriate scooping. For example, at Facility 3:

*“A dedicated green scoop was reported by the chef to be used for portioning protein, a smaller blue scoop for vegetable and carbohydrate portions, and an 89 mL ladle for soft and bite-sized protein or meal (e.g., macaroni and cheese) portions. However, during mealtime, there was no consistency in terms of serving portions and textures”. *

*(Observation)*

Some facilities routinely served fruit puree with breakfast porridge, while others served cut-up fruit platters or fruit puree as desserts at lunch/dinner. In one facility with a mixed level of care, it was consistently observed and reported that residents on TMDs in hospital-level care were not offered texture-modified fruit during the day. Nevertheless, residents on soft and bite-sized diets living in rest-home-level care would have diced fruits for breakfast. It was common for the residents on TMDs to have less than one serving of fresh fruit (150 g) per meal because of the small serving size.

*“They (residents) would usually have sliced peaches or prunes with their porridge (in the rest home dining rooms. But we don’t give those to the pureed (residents) on the other side (hospital-level). Sometimes the kitchen will have strawberry or orange puree, but not very often”. *

*(HA 02)*

*“Commonly, 1/4—1/2 a cup of cut-up fruit was offered to soft and bite-sized diets, and 2–3 tablespoons of fruit purees were offered for pureed and minced and moist diets.” *

*(Observation)*

#### 3.1.3. Food Preferences

The observations and interviews revealed that the majority of residents enjoyed sweet treats, especially ice cream. Although desserts and sweet snacks were provided across facilities, foodservice managers reported the challenge of preparing pureed desserts. Therefore, plain jelly, fruit purees, and ice cream were common substitute dessert options. The Fortisip (a particular brand of ONS produced by Nutricia Pty Ltd., Sydney, Australia) milkshake prepared with strawberry syrup by one facility was favourably consumed by residents. It was commonly consumed in replacement of meals for residents who struggled with TMD meals, particularly pureed. This was also true with the flavoured bottled Fortisip.

Residents who self-fed were observed to favour eating their dessert, rather than their meal. Furthermore, it was observed that some cognitively impaired residents receiving pureed meals would close their mouths while being fed and refuse to eat. It was only when they were offered Fortisip or a dessert, such as ice cream, that they would open their mouths. It was discussed with HAs that sweets were, indeed, a common preference.

*“Sometimes he (a resident on a pureed diet) doesn’t eat anything, but when we give him ice cream, he would eat it”. *

*(HA 05)*

### 3.2. Serving Procedures

#### 3.2.1. Timing of ONS, Desserts, and Drinks

Although ONSs were frequently consumed, there were observed inconsistencies in the serving of ONSs and awareness of consumption by HAs. ONSs were commonly prepared using powdered Fortisip or Ensure. Some foodservice organisations would offer ONS milkshakes for mid-meals, while others simply mixed ONS powder with water to be administrated by HAs at mealtimes or by nurses during medication rounds. Staff reported that food was always trialled first, then ONSs were offered; however, not all HAs seemed to adhere to the protocol.

*“Some residents were fed ONS first, while others were fed ONS with the meal in between mouthfuls”. *

*(Observation)*

HAs were more proficient in monitoring ONS consumption when offering meals in comparison to when nurses administrated them with medications. It was observed that when nurses changed shifts they were unaware of the residents’ intake throughout the day due to insufficient documentation. This was also observed to be influenced by residents who prolonged consumption due to taking frequent sips throughout the day rather than consuming all at once. The same issues were also noted with drinks and desserts. As reported by a HAs and from our observations, a pureed diet resident consistently drank a jug of orange juice throughout the day, which influenced meal consumption. Similarly, most facilities offered residents dessert with meals, rather than after. This was observed to be for the convenience of HAs while serving meals. Staff reported that for some residents with small appetites they would skip offering snacks so they could focus on eating their meals instead.

For those who required thickened fluids, drinks were often only offered at mealtimes. Residents often did not have the access to thickening powder and were unable to prepare their own drinks. There were also no pre-thickened drinks available.

*“Staff served the meals first, then prepare a jar of thickened beverages and poured a glass to those who required thickened fluids. During mid-meals, staff brought a can of thickening powder or a bottle of thickening gel on a trolley and added to pre-prepared coffee/tea”. *

*(Observation)*

*“We usually make the (thickened) drinks at mealtimes. They (residents) do not often ask for drinks. We feed the drinks with foods”. *

*(HA 04)*

#### 3.2.2. Feeding Assistance

Although feeding assistance promoted intake, it was apparent that staff were not always available to assist residents with feeding. Smaller sites were observed to be more organised and settled, as they had more HAs available to provide feeding assistance and cater to residents compared to the larger sites. The staff from a large site reported that there used to be a floater who assisted feeding; however, they were no longer available due to the cost.

Furthermore, there was a lack of a standardised feeding routine to facilitate feeding efficiency. It was common for residents to be left waiting for food while staff were assisting other residents. This resulted in the residents’ meals becoming cold. It was also observed that residents who were fed immediately complained of hot food, as staff did not check the temperature before feeding. This was identified to be an issue upon conversation with a nurse at one of the facilities:

*“Some residents complain that the mash scoop is hot but other meal portions are cold”. *

*(Nurse 02)*

Despite the challenge of catering for multiple residents, particularly in hospital- and dementia-level dining rooms, verbal encouragement was frequently observed across all facilities. Common verbal prompts were: “well done!”, “very good, almost finished!”, “open your mouth, please”, “have some more!”, “try this one”, or calling the resident by name to encourage intake. Overall, it was observed that all aged care residents required encouragement to eat, regardless of feeding requirement. Some residents were also observed to prefer being fed, despite not requiring feeding assistance, while others preferred to self-feed. There was an improved social interaction and a reduced amount of rushed feeding when staff sat with residents rather than standing and feeding between residents.

Most staff reported having received some form of nutrition education in regard to feeding and mealtime assistance. Nonetheless, inconsistent practices involving mixing meal components during feeding were observed at all sites.

#### 3.2.3. Labelling and Serving

Although three levels of TMDs were provided, food/drink items were not specified on the menu for each level but were only listed as “texture modified diet”. In addition, some days the food prepared by the kitchen was inconsistent with the menus as a result of using convenient ingredients. Therefore, the staff relied on the meal labels for delivery and serving. Inconsistent labelling and coverings of TMDs was a common finding across the facilities. Meals were served uncovered without clear labels, causing confusion among the staff during mealtimes.

Only one facility placed IDDSI colour-coded labels on the cling films coved on the plates, and plates were then placed in a ScanBox for delivery. Two facilities wrote residents’ names or meal textures on the cling films with a marker. The labels helped staff easily identify different textured meals. At the other two facilities, meals were rarely labelled or not clearly labelled and it was left to the staff’s knowledge to identify which meal belonged to which resident. For example, at Facility 3:

*“Pureed and minced and moist meals were sometimes observed to be tin-foiled and labelled by a marker and taken via trolley from the kitchen to dining room. However, the foil cover was occasionally removed by the chef in the kitchen when adding portions, rather than by the HAs during meal service”. *

*(Observation)*

When there were no labels or unclear labels, this created an unorganised meal service and delayed feeding. The TMD meals were served from labelled bulk containers at Facility 3, where the fresh cooked meals were plated and served in the dining room. Despite being labelled with IDDSI stickers on the container covers, staff were still confused once the lids were taken off as meals were served onto plates. Meals were also observed to be inconsistently covered upon serving unless meals were taken to residents’ rooms.

### 3.3. Dining Environment

#### 3.3.1. Personal Space

It was observed that the majority of residents finished their meals when they ate in their rooms compared to the dining room or lounge. This was especially true when staff fed residents in their rooms since feeding was more targeted. HAs appeared to take their time with feeding and typically stayed until the resident had finished before moving on to the next resident. Staff reported it was easier for them to keep track of consumption and provide more individualised care in residents’ rooms.

A few residents on pureed diets indicated their preference to eat in their rooms rather than engage in social dining.

*“I felt very different, like embarrassed when they gave me those soft foods (pureed meals). So, I do not like to go to the dining room”. *

*(Resident 02)*

In the interviews with HAs, it was reported that residents generally felt more comfortable in their rooms, and this was especially relevant to those in hospital-level care. For example, a resident on a soft and bite-sized diet reported that he prefers to eat in his room so he is not distracted, in order to feed himself slowly and prevent choking. The staff also mentioned that particular residents stopped attending the dining rooms after being prescribed TMDs.

#### 3.3.2. Dining Room Setting

In the dining room or lounge, age-appropriate music was played at four facilities. All facilities offered dining rooms and lounges that were open spaces and/or had garden views. Rest-home-level dining rooms were usually smaller than hospital-level dining rooms, with more residents sitting at one table. Some facilities had inconsistent dinnerware, including different sizes of bowls, lipped plates, and normal plates. Some residents ate better with smaller dessert spoons compared to normal tablespoons, as observed and reported by the staff. The staff started cleaning up when half of the residents finished their meals. The main meal typically took one hour, including set-up, serving, and cleaning up. Social interaction was minimal during mealtimes between residents. However, some communication occurred between the staff and residents while feeding.

It was also observed that a loud, unorganised mealtime experience in the dining room or lounge caused delays in eating and/or rushing:

*“Resident’s eating in the dining room… very noisy as staff were serving meals”. *

*(Observation)*

*“Lunch was very disorganised; HAs have to work from memory when serving regular/soft and bite-sized meals as they need to consider resident preferences and whether or not they can tolerate certain foods (they also decide how much food to serve)”. *

*(Observation)*

Despite residents all having regular seats, seating arrangements varied across facilities. Some facilities distributed the residents by meal textures, while others were arranged by mobility level, feeding assistants, or cognitive level. Both HAs and foodservice staff reported that some residents on pureed meals can be influenced by residents on regular meals. This was relevant for cognitively aware residents who typically ate in the dining rooms.

*“I don’t feel good when I see other people are eating different food. I don’t want to be different”. *

*(Resident 04)*

*“He refused to have the puree and wanted the same food as the others. He eats more when he eats by himself in the room”. *

*(Foodservice staff 03)*

## 4. Discussion

To the best of our knowledge, this is the first study to apply a mixed methods design to the investigation of the TMD provision and mealtime challenges in quality, feeding, and environment among TMD consumers in aged care facilities. The current study identified positive practices and several challenges related to foodservice production, serving procedures, and residents’ perception of the dining environment contributing to a less satisfactory mealtime experience. The findings of this study are in agreement with the recent studies that examined the mealtime challenges, menus, and food choices of TMD in aged care facilities [21,43,44,45,46,47]. It is important to note that, despite the recent IDDSI implementations addressing the criteria of safe and nutritious TMDs, further quality improvements on TMD service are still required.

### 4.1. Foodservice Production

The current study used an updated menu audit tool that incorporates the IDDSI standards and covers more components of TMDs [10,45]. Despite all levels of TMDs being offered, the TMD menus had inadequate variability and food choices. Similar results were found in Australian aged care facilities, where the TMD menus offered fewer choices than regular menus and meals adhered poorly to the planned menus [46,47,48]. In Miles et al.’s study, residents on TMDs reported that sweet snacks brought them the most pleasure [44]. We observed this phenomenon during mealtime, including for residents with cognitive impairments. Though residents’ food preferences and eating habits were known to the staff, no alternative menu choices were available. Lacking documentation of resident dietary requirements and mealtime preferences was identified in a previous study [49]. For facilities to capture individual preferences, regular consumer satisfaction surveys should be conducted with residents. This may prevent wasting resources and offering inappropriate food options [43,44,50]. Food waste monitoring can be a useful alternative for identifying residents’ preferences, particularly for those with difficulties expressing themselves [51,52]. Implementing resident-centred menus with sufficient food choices can encourage resident decision making and therefore improve residents’ satisfaction and nutritional status [48,53,54]. Individual dietary management files should be created and kept up-to-date, and the importance of maintaining these files should be made known to all staff.

In addition to the food choices, our study supports the findings of previous studies where the nutrition adequacy of the menus failed to meet all recommendations due to the small and inconsistent portion sizes [10,45]. The results indicate that of all food components audited, only fruit and high-fibre food provisions were less than optimal. Although fresh fruits were available, the uncut fruits were inappropriate for dysphagic residents [45]. Foodservice staff are required to put more effort into selecting suitable fruits and high-fibre foods and blending or mincing them for TMDs. Due to the restriction of food texture, TMD consumers often avoid fibrous food, such as whole grains, husks, raw fruits, and vegetables. It has been reported in a recent review that inadequate fibre intake is a common issue for TMD consumers, in part due to the lack of fibre content offered on TMD menus [22]. The fibre and micronutrients contained in fruits are crucial for older adults. For example, a fruit and fibre-rich porridge was found to be an effective dietary strategy for treating constipation and reducing the use of laxatives in older adults [55]. Pureed lentils and split peas are also good sources of fibre that can be added into mashed potatoes or soup to provide better consistency [56].

Producing palatable TMDs has been a constant challenge for foodservice [8,13,21]. Older adults commonly experience impaired sensitivity of taste and smell, which leads to poor appetite and decreased nutrition intake [57]. The provision of flavour-enhanced and appealing TMDs can compensate for the sensory loss [58]. Residents were more satisfied with moulded TMDs, and foodservice production should consistently match these expectations [21,43,44]. While the positive feedback of moulded TMDs was supported by our study, it should be noted that the residents still found it difficult to identify the food [43]. Furthermore, the absence of TMD menus in dining rooms and serving trays also accounted for the inability to distinguish food. Food scientists are attempting to develop tasty, safe, and nutritious commercially prepared TMDs [59]. Four sites used commercially prepared TMDs, which have gained popularity, as they are nutrient-fortified and comply with IDDSI standards [22,44]. The staff reported that commercial products saved time and evoked less safety concerns. Despite the higher cost of commercial products, savings can be made from reduced staff preparation time and minimised hospitalisations [11,13,60]. Foodservice can implement a single affordable change that would increase residents’ enjoyment and dietary consumption [61].

### 4.2. Serving Procedures

There is mixed guidance in the literature regarding mealtime preferences. Some residents report preferring flexible mealtimes, while others favour fixed mealtimes that provide food security [50,62]. Observations indicated that meals were provided at specific times. Due to the limited staff availability and a high demand for feeding assistance, flexible mealtimes can be costly and overwhelming for staff [27,63]. Residents on TMDs were less active and more likely to be sleeping throughout the day, which resulted in missing the mid-meals or skipping ONS [64]. Considering the high risk of inadequate nutrition intake among TMD consumers, it is important to ensure residents do not skip meals [22]. When residents are not rousable or refuse to consume food at mealtimes, staff should offer the food again on awakening. Another challenge we found was the serving timing and sequence of the ONS, meals, and desserts. Although it is recommended to first offer high-energy foods to those with small appetites, the observations suggested doing so can distract residents from finishing their meals [65]. Previous studies were unable to draw conclusions of the most effective meal patterns [66,67]. In order to determine food choices and feeding sequences, dietitians should be consulted when someone has inadequate oral intake or poor compliance to mealtimes. Residents receiving TMDs have a higher demand for feeding assistance [10,66,68,69]. Despite staff demonstrating positive attitudes towards feeding, feeding assistance was made difficult by limited staff numbers. For example, staff were more likely to stir meals together when under time pressure. Comparing the findings with other studies confirms that inadequate staffing contributes to residents having cold meals and falling asleep [8,25,27]. When mealtime assistance is inadequate, residents may have difficulty completing their meals, resulting in increased psychological stress and reduced oral intake [49]. For facility management and policymakers, attention should be paid to staffing availability and emphasise efficient practices. A previous study suggested mealtime interactions and the familiarisation between staff and residents were more important than having a high number of staff [25]. In order to achieve safe eating, HAs and nurses should be aware of the TMD standards and be able to check that the appropriate food type and consistency were offered [70]. To avoid confusion, TMDs should be labelled with IDDSI colour-coded levels. Alternatively, different coloured plates can be used for each level of TMDs. It should be noted that pureed food should not be given to residents on soft and bite-sized or minced and moist diets out of convenience. A previous study found no association between TMDs and longer mealtime duration [71]. Therefore, staff can pre-arrange designated feeding routines for individuals requiring assistance.

Serving TMDs at their appropriate temperature also increases consumers’ acceptance [43,72]. Temperature has a strong impact on TMDs since a small temperature change can result in texture change [73]. The majority of the facilities in our study used plated meal services to distribute TMDs. Studies have shown that food served from bulk-tray service maintains a better temperature, looks more appealing, feels more home-like than plated meals, and leads to consumer satisfaction [74,75].

### 4.3. Dining Environment

Eating with others and the social atmosphere can have a positive influence on mealtime, though a calm mealtime environment is imperative for older adults [24,27,76,77]. Bennett et al. reported that residents prefer to keep their privacy and eat in their rooms [49]. Our findings reflect those of Watkins et al., where some residents preferred to eat in the dining room, while others liked to eat in their own room [28]. A high demand for staff attention and supervision may explain why residents were often brought into the dining rooms for easier observation. Mealtimes could be rushed, with staff frequently multitasking in the dining rooms, particularly in hospital-level care. It is recommended that staff should attempt to provide timely feeding assistance and uninterrupted focus at mealtimes [70]. Despite the dining rooms being appropriately arranged, the loud noises and busy environment may cause residents to feel distracted and stressed [27]. In contrast, smaller dining rooms with fewer residents or residents’ own rooms enabled more individualised care and targeted feeding assistance. The mealtime experience can be a personal choice determined by the state of mind, health conditions, and personality rather than environmental factors [78]. Therefore, aged care facilities should encourage residents to choose their preferred dining location and motivate independence in eating by offering space and self-feeding dinnerware. By empowering residents with more mealtime control, they would feel more comfort and pleasure, which can have a positive impact on their quality of life [25,50].

### 4.4. Limitations

By using a conventional content analysis approach, this study was able to gather information directly through observing and interviewing relevant personnel without imposing preconceived themes and therefore contributes robust insights to future TMD research in aged care [34]. However, it is difficult to draw general conclusions from this study because of its exploratory nature. The results may only apply to the aged care facilitates observed and to residents requiring TMDs. As a result of convenience sampling, the facilities that participated in the study may be more confident and enthusiastic in providing quality care. Other facilities may experience greater challenges. Future research should conduct consistent observations with larger samples and over different days of the week. Finally, we cannot rule out the possibility that staff may have altered their usual behaviour and practices since they were being observed.

## 5. Conclusions

The results from this study provide an in-depth observation of the mealtime practice of aged care residents consuming TMDs, including the quality of their TMD menus, mealtime assistance, and their dining room environment. In order to provide a safe and pleasurable mealtime experience that maximises residents’ nutritional intake and psychosocial needs, aged care facilities need to focus on mealtime customisation from the TMD production in kitchens to feeding in the dining rooms. Mealtime challenges involve multiple perspectives; therefore, they need to be tackled by a multi-disciplinary team. Aged care should consider empowering dietitians’ engagement in foodservice management and incorporate routine TMD audits into practice. Dietitians should work closely with foodservice to standardise the meal portions and offer more suitable food varieties for individual preferences. Regular team meetings are important for staff communication and information updates. Facilities should consider offering continuous training to enhance staff awareness of mealtime issues. Although the IDDSI has been implemented, there is a lack of clinical standardisations targeting mealtime management. For foodservice to offer nutritious and safe TMDs, there are several challenges to overcome. Policymakers should work with health professionals and researchers to develop guidelines that can facilitate mealtime quality in aged care facilities.

## Figures and Tables

**Table 1:** Aged care texture-modified diet menu audit results: compliance with each subsection of Dietitian NZ foodservice and nutrition audit recommendations.

Outcomes (Number of Objectives Measured in Each Category) *	Facility 1 % (*n*)	Facility 2 % (*n*)	Facility 3 % (*n*)	Facility 4 % (*n*)	Facility 5 % (*n*)
**Overall food quantity and nutrition adequacy (26)**	74 (19)	74 (19)	74 (19)	74 (19)	74 (19)
Nutrition quality (12)	92 (11) **	92 (11)	92 (11)	92 (11)	92 (11)
Meat/Alternatives (3)	100 (3)	100 (3)	100 (3)	100 (3)	100 (3)
Dairy (1)	100 (1)	100 (1)	100 (1)	100 (1)	100 (1)
Carbohydrates (1)	0 (1)	0 (1)	0 (1)	0 (1)	0 (1)
Fibre (1)	0 (1)	0 (1)	0 (1)	0 (1)	0 (1)
Fruit/vegetables (3)	67 (2)	67 (2)	67 (2)	67 (2)	67 (2)
Snacks (1)	100 (1)	100 (1)	100 (1)	100 (1)	100 (1)
Fluids (2)	0 (0)	0 (0)	0 (0)	0 (0)	0 (0)
TMD menu quality (2)	50 (1)	50 (1)	50 (1)	50 (1)	50 (1)
**Overall foodservice and kitchen-related standards (51)**	86 (44)	86 (44)	88 (45)	80 (41)	88 (45)
Dining environment (11)	73 (8)	73 (8)	82 (9)	73 (8)	82 (9)
Actioning of policies and procedures (3)	100 (3)	100 (3)	100 (3)	100 (3)	100 (3)
Foodservice practices (8)	88 (7)	88 (7)	88 (7)	75 (6)	88 (7)
Meal quality (2)	100 (2)	100 (2)	100 (2)	100 (2)	100 (2)
Hydration (2)	100 (2)	100 (2)	100 (2)	100 (2)	100 (2)
Menu (6)	83 (5)	83 (5)	83 (5)	83 (5)	83 (5)
Oral nutritional supplements (4)	100 (4)	100 (4)	100 (4)	75 (3)	100 (4)
Portion size (5)	80 (4)	80 (4)	80 (4)	80 (4)	80 (4)
TMD compliance to IDDSI (10)	90 (9)	90 (9)	90 (9)	80 (8)	90 (9)
**Overall clinic-related standards (16)**	81 (13)	81 (13)	81 (13)	81 (13)	81 (13)
Clinical practices (10)	90 (9)	90 (9)	90 (9)	90 (9)	90 (9)
IDDSI practice (4)	50 (2)	50 (2)	50 (2)	50 (2)	50 (2)
Training (2)	100 (2)	100 (2)	100 (2)	100 (2)	100 (2)
**Total compliance (93)**	82 (76)	82 (76)	83 (77)	78 (73)	83 (77)

Note. TMD = Texture-Modified Diet; IDDSI = International Dysphagia Diet Standardisation Initiative; * Outcomes are listed according to objectives and subsections in the Dietitian NZ foodservice and nutrition audit sheet [33]. ** Results are presented as percentage of compliance (number of compliant objectives).

**Table 2:** Non-compliant objectives in texture-modified menus identified via menu audits in five aged care facilities, ranked by compliance failure frequency.

Subsections *	Non-Compliant Objectives	Frequency (*n*/5) **
**Food quantity and nutrition adequacy**	1. Menu did not specify all IDDSI textures for meals and snacks	5
	2. Menu did not specify all food textures for each meal and snack (e.g., bread is not suitable for TMDs)	5
	3. Inadequate provision of high-fibre whole-grain foods throughout the day	5
	4. Inadequate high-fibre food offered at every meal and snack	5
	5. Inadequate fresh fruit was offered daily	5
	6. Fluid varieties were not specified on the menu	5
	7. Fluid provision times were not specified	5
Foodservice and kitchen-related standards	1. No second helping was offered to residents who finished their meals	5
	2. Jelly was not served with high-protein/milk-based accompaniments	5
	3. Food wastage was not monitored systematically	5
	4. Inadequate portions of fruits were served over the day	5
	5. Texture-modified meals were stirred together during feeding	5
	6. TMD menus were not displayed in the dining room	4
	7. Desserts were given before the main meal was finished	3
	8. When residents were sleeping/sleepy during mealtime, the risk of malnutrition was not addressed to the nursing team	1
	9. Food fortification strategies were not in place	1
	10. Foodservice did not have high-protein, high-energy drinks available in addition to oral nutritional supplements	1
	11. Level 4 and level 5 TMDs were not fortified	1
Clinic-related standards	1. Nutrition support was not always initiated for at-risk residents (e.g., wound and pressure injuries, bowel issues, respiratory disease, frequent UTIs, etc.)	5
	2. Not all healthcare assistants were able to demonstrate fluid tests for each level of fluid thickness	5
	3. Daily fluid monitoring form was not in place for residents on thickened fluids	5

Note. TMD = Texture-Modified Diet; IDDSI = International Dysphagia Diet Standardisation Initiative. * Subsections were the summaries of all objectives measured in the audit and are listed according to the Dietitian NZ foodservice and nutrition audit sheet [33]. ** Frequency is presented as the number of non-compliant facilities out of the total of five facilities.

## Data Availability

The datasets used and/or analysed during the current study are available from the corresponding author on reasonable request.

## Supplementary Materials

The following are available online at https://www.mdpi.com/article/10.3390/geriatrics7030067/s1, File S1: Menu audit tool adapted from Dietitian NZ foodservice and nutrition audit sheet, File S2: Mealtime observation form.
