# Supplementary material for: Exploring Meal Provision and Mealtime Challenges for Aged Care Residents Consuming Texture-Modified Diets: A Mixed Methods Study
Source: Geriatrics (Basel). 2022 Jun 15;7(3):67. doi: 10.3390/geriatrics7030067 (PMC9222299; doi:10.3390/geriatrics7030067)
Supplement: Supplementary file 1 [file geriatrics-07-00067-s001.zip › Supplementary File S1 - menu audit tool.pdf]

Supplementary File S1. Menu Audit tool adapted from Dietitian NZ foodservice and nutrition audit sheet

FACILITY INFORMATION

**Organisation Name**	
**Level/type of care provided (current number of residents)**	Rest home Hospital Dementia<br>Service Apartments Independent Living Extra Services
**Total number of beds**	
**Current occupancy**	
**Ethnicity (cultural diversity of your residents)**	
**Age of clients**	Average (approx.) Age range
**Number of residents**	Male Female
**Texture Modified Dietary Requirements (current number of residents)**	Easy to Chew: Soft & Bite: Minced & Moist:<br>Pureed: Thickened fluids:
**Number of residents are on supplements**	
**Time/Meals of supplementation provided**	☐Breakfast ☐Morning tea ☐Lunch ☐Afternoon tea ☐Dinner ☐Supper
Criteria of providing supplements (e.g. resident preference, clinician/family required, observation of resident decreased oral intake)	
**Date of audit report**	
**FOOD SERVICE INFORMATION**	
**Type of food service**	☐Cook fresh ☐Cook-chill ☐Cook-freeze ☐Commercial pre-prepared ☐Other_________
**Type of Texture-modified diets provided**	☐Made in-house ☐Commercial pre-prepared ☐Mixed
**Fortification of Texture-modified meals**	Y/N Details of fortification________________________________
**Is a cycle menu used and if so, how many weeks per cycle?**	Y/N Number of weeks_____
**Is there a separate winter & summer cycle or one menu for the year?**	winter and summer cycle **OR** one menu for the year
**Main meal served (please indicate)**	Midday **OR** Evening
**Mealtimes**	Breakfast: Lunch: Dinner:
**Mid mealtimes**	Morning tea: Afternoon tea: Supper:

No.	Objective	Standard	Compliance (Y/N)	Evidence

SECTION A: Food quantity and nutrition adequacy				
1.01	Overall Menu	Menu fatigue by residents is minimised through length of menu cycle and variations between seasonal menus.		
1.02	Overall Menu	The menu must offer 3 meals and 3 between-meal snacks		
1.03	Overall Menu	The menu provides sufficient variety within each of the food groups.		
1.04	Overall Menu	The menu offers variety within meal types.		
1.05	Overall Menu	The menu reflects seasonal variations in fruit and vegetables.		
1.06	Overall Menu	The menu is not planned specifically to use leftovers.		
1.07	Overall Menu	The menu should reflect the generational, religious, and cultural food preferences of the residents.		
1.08	Overall Menu	Main meal ingredients are not repeated within the same day or on consecutive days		
1.09	Overall Menu	The meals as planned are likely to be visually appealing and allows for good presentation		
1.10	Overall Menu	There are no generic diabetic menus, nor diabetic options on the standard menu ('diabetic diets' or modifications should only be part of individualised dietitian assessed nutrition care plans).		
1.11	Overall Menu	There is provision for specialised diets on the menu where required.		
1.12	Overall Menu	The menu clearly specifies all IDDSI textures for each meal and snack		
1.13	Meat / Alternatives	At least 2 serves of meat or meat alternatives are offered over the course of the day.		
1.14	Meat / Alternatives	A high protein food must be offered at each meal.		
1.15	Meat / Alternatives	Fish (including canned) is included at least twice a week.		
1.16	Milk & Milk Products	At least 3 servings of milk or milk products are offered on the menu each day (excluding incidental use in beverages or soups and excluding cream).		
1.17	Breads, grains, cereals and carbohydrates	High fibre wholegrain products and foods are available throughout the day.		
1.18	Fibre	At least one high fibre food should be offered at every meal and at least one snack. (foods with more than 3g/100g of fibre)		
1.19	Fruit / Vegetables	At least 3 servings of vegetables are offered each day.		
1.20	Fruit / Vegetables	At least 2 servings of fruit are offered each day.		
1.21	Fruit / Vegetables	At least 1 serving of uncooked fruit is offered daily.		
1.22	Snacks	A high protein option should be available at each snack.		
1.23	Fluids	The menu specifies a variety of fluids to support fluid intake, e.g. range of flavours and hot and cold options.		
1.24	Fluids	The menu specifies at least 8 opportunities per day for fluid intake.		

1.25	Texture Modified	The menu clearly specifies all food textures in line with the IDDSI framework for each meal and snack.		
1.26	Texture Modified	Foods offered on the texture modified menu allow for a visually appealing meal		
**SECTION B: Foodservice & kitchen related standards**				
**2.05**	Dining Environment	The dining room provides a comfortable dining environment that supports food intake.<br>Includes; noise, lighting, odours, space, decorations, table settings, distractions etc.		
**2.06**	Dining Environment	A menu is on display in the dining room and details the meals for today including any texture modified diets.		
**2.07**	Dining Environment	Where required, staff explain to residents what their meal is.		
**2.08**	Dining Environment	Residents who finish their meals are offered a second helping		
**2.09**	Dining Environment	Desserts are given after the main meal is finished, (to avoid overwhelming/rushing over mealtime and to prevent desserts being eaten instead of eating both the main and dessert)		
**2.10**	Dining Environment	Residents who decline the main meal are offered suitable alternatives. Within reason dislikes are catered for.		
**2.11**	Dining Environment	Staff attempt multiple solutions for residents who have not settled for the meal or who are not eating.		
**2.12**	Dining Environment	Specialised cutlery, cups and plates are used where necessary to support self-feeding		
**2.13**	Dining Environment	Supervision is provided in all dining rooms and assistance is provided for residents needing help at mealtime, including feeding assistance.		
**2.14**	Dining Environment	If there are residents sleeping or sleepy during mealtime, has this been highlighted with the nursing team as a risk for malnutrition?		
**2.15**	Dining Environment	Staff are able to recall when asked some of the common signs and symptoms of dysphagia during eating and drinking.		
**2.16**	Foodservice Practices	Home baking is incorporated in the menu at least daily		
**2.17**	Foodservice Practices	Iodised salt is used.		
**2.18**	Foodservice Practices	Commercial soup powders are used only as a base		

**2.19**	Foodservice Practices	As a dessert or snack, Jelly is only served if it includes a high protein/milk based accompaniment		
**2.20**	Foodservice Practices	Cream is used occasionally, not as a replacement for other high calcium and protein dairy accompaniments.		
**2.21**	Foodservice Practices	Artificial sweeteners are NOT routinely added although are available on site only for resident preference.		
**2.22**	Foodservice Practices	Sugar-free cordials and drinks are available if clinically indicated.		
**2.23**	Foodservice Practices	There is no separate 'diabetic diet' options prepared (unless required for an individual diet plan developed with input from the clinical dietitian)		
**2.24**	Actioning of Policies & Procedures	Food fortification strategies offer effective solutions to increase the protein and/or energy content of foods, drinks and snacks.		
**2.25**	Meal quality	The presentation of the meals is good and encourages food intake.		
**2.26**	Meal quality	Complete a quality check. Is the quality of the meal (all courses) acceptable.		
**2.27**	Hydration	It is observed that residents are supported or encouraged to drink during the mealtimes and beverage rounds.		
**2.28**	Hydration	A range of suitable beverages is available during beverage rounds or at beverage stations.		
**2.29**	Menu	The menu used on site matches the dietitian reviewed menu		
**2.30**	Menu	The meals provided match the menu used on site. The menu is routinely followed by the kitchen staff.		
**2.31**	Menu	There is evidence that menu items not enjoyed by the majority of the residents are either removed from the menu or an alternative is offered.		
**2.32**	Menu	The meal service (foods and drinks) is appropriate for the cultural preferences of the residents being served.		
**2.33**	Menu	Feedback from residents suggests that overall the residents enjoy the meals provided.		
**2.34**	Menu	There is a system to monitor high food wastage, (suggesting low food intake) to review the meal option and take corrective action.		

**2.35**	Actioning of Policies & Procedures	The up-to-date policies for nutrition, foodservice and hydration are accessible in the kitchen.		
**2.36**	Actioning of Policies & Procedures	There is an effective system in the kitchen to ensure all residents requiring a high protein high energy diet recieve the foods and drinks in accordance with the individual resident nutrition care plan from the clinical team or dietitian.		
**2.37**	Oral Nutritional Supplements	Oral nutritional supplements are routinely provided between meals to support food first and allow ONS to provide additional protein and calories. (Exception if ONS prescribed on the medication chart specifically at mealtimes)		
**2.38**	Oral Nutritional Supplements	High protein / high energy oral nutrition supplements are provided as prescribed and are prepared in accordance to the dietitian's instructions (or if no specific instructions by the manufacturer's instructions).		
**2.39**	Oral Nutritional Supplements	High protein high energy drinks, prepared by the food service are available in addition to, or instead of ONS		
**2.40**	Oral Nutritional Supplements	Recipes for HPHE drinks to be prepared on site are available and are followed.		
**2.41**	Portion size	At least 80g cooked meat (or 135g legumes) per serving is served at the main meal		
**2.42**	Portion size	The main option(s) served at the secondary meal provides a protein source (equivalent to at least 15g protein per serving).<br><br>*This does not include other meal components served with the meal such as soups, sandwiches, desserts etc.*		
**2.43**	Portion size	At least 3 servings of vegetables are served over the day (serving equivalent to at least 60g - 80g) (excluding potato)		
**2.44**	Portion size	At least 2 servings of fruits are served over the day (serving equivalent to one piece of fresh fruit, 2 x small fruit or 1/2 cups stewed fruit (135g)		
**2.45**	Portion size	Residents receiving serving sizes less than recommended are identified and additional nutrition strategies are in place to help meet nutritional needs.		
**2.46**	Texture Modified	Texture modified meals are prepared in accordance with the International Dysphagia Diet Standardisation initiative (IDDSI) Framework: (easy to chew, soft & bite sized, minced & moist, pureed)		
**2.47**	Texture Modified	Thickened fluids are prepared in accordance with IDDSI guidelines.		
**2.48**	Texture Modified	If there is sauce or gravy served with a texture modified meal, it is suitable for the meal and dysphagia requirements.		
**2.49**	Texture Modified	Those on an IDDSI food or fluid prescription are provided the correct meal texture or fluid thickness.		

**2.50**	Texture Modified	The presentation of the texture modified meals is good.		
**2.51**	Texture Modified	The IDDSI standards are displayed in the kitchen		
**2.52**	Texture Modified	Any staff involved in texture modified meal preparation is able to demonstrate IDDSI testing for each of the soft & bite-sized, minced and moist, and puree textures.		
**2.53**	Texture Modified	Texture modified meals are not stirred together by supporting staff during feeding.		
**2.54**	Texture Modified	Level 3 Liquidised, Level 4 Puree and Level 5 Minced Moist meals are fortified with added energy and/or protein.		
**2.55**	Texture Modified	A variety of thickened fluids are offered over the day.		
**SECTION B: Clinical related standards**				
**2.56**	Clinical Practices	The clinical team monitor weights at least monthly and have highlighted residents with a pattern of unwanted weight loss		
**2.57**	Clinical Practices	Nutrition screening for malnutrition using a validated screening tool is conducted for all residents. The clinical team have a record of residents identified as malnourished or at risk of malnutrition.		
**2.58**	Clinical Practices	The malnutrition screening tool is completed correctly and accurately.		
**2.59**	Clinical Practices	The nutrition policy for malnutrition and unwanted weight loss is followed for any resident identified as being malnourished or at risk of malnutrition		
**2.60**	Clinical Practices	Further action is taken for any resident with on-going weight loss and/or poor food intake despite nutrition intervention and this includes referral to a dietitian.		
**2.61**	Clinical practices	Are there any residents with the following conditions where nutrition intervention can assist with improving outcomes?<br>- wounds and pressure injuries<br>- constipation / diarrhoea / bowel issues<br>- respiratory disease, COPD<br>- GORD<br>- recent surgery<br>- recent hospital admission<br>- fractures<br>- frequent UTIs		
**2.62**	Clinical practices	For residents with any of the above conditions has additional nutrition support been initiated. Has the clinical dietitian had input?		

**2.63**	Clinical Practices	Nutrition care plans are individualised and comprehensive.		
**2.64**	Clinical Practices	Restrictive diets are only implemented if clinically indicated.		
**2.65**	Clinical Practices	All residents requiring or requesting restrictive or specialised diets from the facility, who are not being provided meals from a dietitian audited menu, should have their diets reviewed by a dietitian.		
**2.67**	Texture Modified	Resident Food/Nutrition/Dietary Forms have IDDSI texture prescriptions as options for food AND for fluid and a food texture and fluid prescription is indicated for all residents.		
**2.68**	Texture Modified	Any staff involved in the preparation of thickened fluids is able to demonstrate safety testing for each level of fluid thickness.		
**2.69**	Texture Modified	The IDDSI standards are easily accessible to all staff in the facility.		
**2.70**	Texture Modified	There is a daily fluid monitoring form in place for all residents on thickened fluid requirement - and there is evidence that hydration needs are being met as much as practicable.		
**2.71**	Training	Kitchen staff, nursing staff and caregivers have received training in the past two years, by a dietitian, on the specialised diets required at the site.		
**2.72**	Training	Training on basic nutrition and hydration for older adults in aged care facilities has been provided by a dietitian in the past two years to all staff including kitchen staff, caregivers and nursing staff.		

Note. 2.01-2.04 were questions specifically for dementia unit and were excluded because it was not included in this study.

SAMPLE MEAL PORTION SIZE AUDIT RESULTS					
**Meal component**	**Description**	**Weight**			
		**Easy to chew**	**Soft & Bite**	**Minced & Moist**	**Pureed**
**Meat**					
**Potato**					
**Rice/pasta**					
**Vegetables**					
**Fruits**					
**Sauce/gravy**					
**Dessert (dairy based)**					
**Ice cream**					
**Dessert (cake/pudding)**					
**Dessert (fruit based)**					
**Custard accompaniment**					
**Cream**					
**Snack**					
**Drinks**					
**Fortification**					

References:

1. Dietitians NZ.Menu audit aged care facilities[Internet]. 2020.https://www.acefoods.co.nz/files/119/file/MENU-AUDIT. Accessed 08 May, 2021.
2. New Zealand Ministry of Health. (2010). Food and Nutrition Guidelines for Healthy Older New Zealanders, A Background Paper.
3. Dietitian Association of Australia. Menu review audit tool for aged care homes [Internet]. 2016.https://daa.asn.au/resource/daa-menu-audit-tool-for-aged-care-homes/. Accessed 29 March 2019.
