# Supplementary material for: Exploring Meal Provision and Mealtime Challenges for Aged Care Residents Consuming Texture-Modified Diets: A Mixed Methods Study
Source: Geriatrics (Basel). 2022 Jun 15;7(3):67. doi: 10.3390/geriatrics7030067 (PMC9222299; doi:10.3390/geriatrics7030067)
Supplement: Supplementary file 1 [file geriatrics-07-00067-s001.zip › Supplementary File S2 - mealtime observation form.pdf]

Mealtime Observation

Date:<br>Mealtime: BF L D	Reviewed by:<br>Facility:

No. of residents on TMDs:	EC7	SB6	MM5	PU4	LQ3

No. of residents on thickened fluids:	EX4	MO3	MT2	ST1

No. of residents required assistance:	Set up	Cut up	Feeding

No. of assistant staff:	Caregivers	Nurses	Others (e.g. family)

Completion of the meals<br>(No. of residents)	All	3/4	1/2	1/4	None

Time to complete meal<br>(No. of residents)	<20 min	20-29 min	30-39 min	≥40 min

No. of residents on ONS:	Describe what was left in the plate
Did the texture/consistency change prior eating?<br>Y / N	
Did the food/drink use IDDSI label?<br>Y / N	Describe the dinning environment (e.g. distractions, music, communications)
Did staff interact with residents?<br>Y / N	
Was the meal covered and the temperature preserved before consumption?<br>Y / N	Describe the assistance provided by the staff
Did staff check the food was correctly delivered?<br>Y / N	Other notes

Photos attached

List of Abbreviation used in the form

BF- breakfast; L – Lunch; D – Dinner

TMD – texture modified diet; ONS – oral nutritional supplement; IDDSI – International dysphagia diet standardisation initiative

Levels of texture modified diets: EC 7 – level 7 easy to chew; SB6 – level 6 soft and bite-sized; MM5 – level 5 minced and moist; PU4 – level 4 pureed; LQ3 – level 3 liquidised

Levels of thickened fluids: EX 4 – level 4 extremely thickened; MO3 – level 3 moderately thickened; MT2 – level 2 mildly thickened; ST1 – level 1 slightly thickened
